# 

**Published:** 1900-02

**Authors:** 


					﻿SUBBCaiPTION 3100.
ATLANTA PUBLISHED MONTHLY.
JOURNAL-RECORD
OF MEDICINE.
Successor to Atlanta Hedical and Surgical Journal, Established 1855,
and Southern fledical Record, Established 1870.
Vol. I.	Atlanta, Ga., February^ 1900.	No. 11.
				

